# *Pnpla5*-knockout rats exhibit reduced expression levels of proteins involved in steroid metabolism and wound healing compared to wild-type rats

**DOI:** 10.1186/s12864-022-08835-8

**Published:** 2022-08-12

**Authors:** Zhi-Guo Liu, Yan-Qing Hu, Kui Li, Yu-Lian Mu, Tian-Wen Wu

**Affiliations:** 1 State Key Laboratory of Animal Nutrition, Key Laboratory of Animal Genetics Breeding and Reproduction of Ministry of Agriculture and Rural Affairs of China, Institute of Animal Sciences, Chinese Academy of Agricultural Sciences, Beijing, 100193 China; 2 Genome Analysis Laboratory of the Ministry of Agriculture and Rural Affairs, Agricultural Genomics Institute at Shenzhen, Chinese Academy of Agricultural Sciences, Shenzhen, 518120 China

**Keywords:** *Pnpla5*, *Pnpla5*-knockout rats, Apoptosis, iTRAQ, Wound healing, Steroid metabolism, Membrane attack complex

## Abstract

**Background:**

Patatin-like phospholipase domain containing 5 (PNPLA5) is a newly-discovered lipase. Although the PNPLA family plays critical roles in diverse biological processes, the biological functions of PNPLA5 mostly unknown. We previously found that the deletion of *Pnpla5* in rats causes a variety of phenotypic abnormalities. In this study, we further explored the effects of *Pnpla5* knockout (KO) on male rats.

**Results:**

The body weight and testicular or epididymal tissue weight of three to six 3-month-old *Pnpla5* KO or wild-type (WT) male Sprague–Dawley rats were measured. The protein expression levels were also measured via western blotting and iTRAQ (isobaric tags for relative and absolute quantitation) analyses. No significant difference between *Pnpla5* KO and WT rats, regarding body weight, testicular or epididymal tissue weight, or hormone levels, were found. However, the relative testicular tissue weight of the KO (*Pnpla5*^*−/−*^) rats was higher (*P* < 0.05) than that of WT rats. Significant increases in apoptotic cells numbers (*P* < 0.001) and BAX and Caspase-9 expression levels were observed in the testicular tissue of *Pnpla5*^*−/−*^ rats. Moreover, iTRAQ analysis revealed that the levels of proteins involved in steroid metabolism and wound healing were significantly decreased in *Pnpla5*^*−/−*^ rats.

**Conclusion:**

This study revealed that *Pnpla5* knockout induced apoptosis in rat testes. We also ascertained that *Pnpla5* plays an important role in lipid metabolism, wound healing, and affects reproductive organs negatively, providing new target genes and pathways that can be analyzed to unravel the biological function of *Pnpla5*.

**Supplementary Information:**

The online version contains supplementary material available at 10.1186/s12864-022-08835-8.

## Background

The patatin-like phospholipase domain-containing (PNPLA) protein family is a newly discovered type of lipases [1]. Ten PNPLA proteins have been identified and characterized in the human genome [2]. The biochemical functions and underlying molecular mechanism of *Pnpla5* remain elusive. Although most tissues of rats [3], mice, and human [4] contain low levels of PNPLA5, expression patterns significantly vary among different species. In rats, the mRNA expression of *Pnpla5* is abundant in the skin, testes, epididymis, uterus, and ovaries [3], while in mice, *Pnpla5* is highly expressed in the lungs, epididymis and brown adipose tissues [4]. The expression pattern of murine *Pnpla5* is highly correlated with that of *Pnpla3*, which located upstream of the *Pnpla5* gene in the mouse genome. Both these genes are inhibited by fasting, up-regulated in the process of adipocyte differentiation and highly activated in the liver of obese (*ob/ob*) mice [4]. Furthermore, *Pnpla5* mRNA expression level increases in *Pnpla*3 KO mice, and it can be up-regulated to reach a level that equates untampered *Pnpla3* level [5], when high sucrose lipogenic diets are administered. This indicates that, in mice, *Pnpla5* partially compensate for the biological deficiencies caused by the absence of *Pnpla*3.

PNPLA5 exhibits triglyceride (TG) hydrolase activity [6, 7], further, the over expression of *Pnpla5* decreases the cellular TG content [4]. Whole-exome sequencing in human also revealed that *Pnpla5* is associated with low density lipoprotein cholesterol (LDL-C) [8]. In our previous study, we bred *Pnpla5* KO (*Pnpla5*^*−/−*^) Sprague–Dawley (SD) rats [3] and found that they have increased serum total cholesterol (TC), TG and high density lipoprotein cholesterol (HDL-C) levels, but reduced LDL-C level. Interestingly, we also found that *Pnpla5*^*−/−*^ rats exhibited abnormal bleeding [3] and reduced fertility [9]. These findings imply that *Pnpla5* not only plays a key role in lipid metabolism, but also affects the fertility of male rats. To further explore the biological functions of *Pnpla5*, the body weight, testicular and epididymal tissue weight, and reproductive hormone levels of *Pnpla5*^*−/−*^ male rats were measured. We detected germ cell apoptosis in rat testicular tissues using terminal deoxynucleotidyl transferase-mediated dUTP nick end labeling (TUNEL) and western blotting analyses. We also performed iTRAQ (Isobaric tags for relative and absolute quantitation) proteomic analysis of testes to identify target genes and pathways that may be regulated by *Pnpla5.*

## Results

### Knockout of *Pnpla5* does not influence the body weight, reproductive organs weight or serum hormone levels of rats

**Fig. 1:**
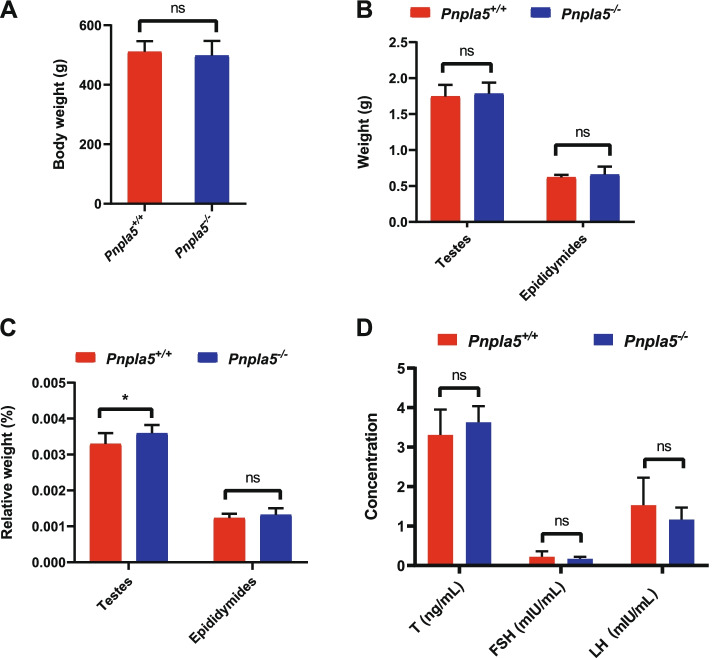
The body weight, testicular and epididymal tissue weight and serum hormone levels of *Pnpla5*^*−/−*^ and *Pnpla5*^+*/*+^ male rats. **A** No significant differences in the body weight between *Pnpla5*^*−/−*^ and *Pnpla5*^+*/*+^ male rats. *P* = 0.49. **B** No significant differences in the testes (*P* = 0.58) and epididymis (*P* = 0.31) weight between *Pnpla5*^*−/−*^ and *Pnpla5*^+*/*+^ male rats. **C** Significant differences in the relative weight of testicular tissue (*P* = 0.02) but not epididymal tissue (*P* = 0.20) were observed * P < 0.05. **D** No significant differences in serum hormone levels between *Pnpla5*^*−/−*^ and *Pnpla5*^+*/*+^ male rats. T: Testosterone (*P* = 0.16). FSH: Follicle-stimulating hormone (*P* = 0.43). LH: Luteinizing hormone (*P* = 0.11). ns: not statistically significant. All data are presented as the means ± standard deviation (SD)

*Pnpla5* plays an important role in lipid metabolism, and its deletion causes disorders of lipid metabolism in rats [3]. Lipid metabolism plays an important role in the reproductive organ-development and spermatogenesis. Vendramini et al. (2014) found that obesity could cause a reduction in testicular weight in male rats [10], and high-fat diet administration alters the testicular morphology of rats [11]. We also reported that *Pnpla5* knockout significantly reduce the fertility of male rats and the curvilinear velocity of their sperm [9]*.* Therefore, we assessed the body and reproductive organs (testis and epididymis) weight, and serum hormone levels of *Pnpla5*^*−/−*^ and WT (*Pnpla5*^+*/*^)^+^ male rats (Fig. 1).

The rats exhibited no significant differences (*P* = 0.49) in their body weight (Fig. 1A). Although the testicular and epididymal weights of *Pnpla5*^*−/−*^ rats increased compared to the control rats, the differences were not significant (*P* = 0.58 for testes, *P* = 0.31 for epididymis, Fig. 1B). We also calculated the relative weight of testicular and epididymal tissues, i.e., the tissue to body weight ratio. In *Pnpla5*^*−/−*^ rats, the relative testicular weights increased significantly (*P* = 0.02), but the relative epididymal weights were not significantly different (*P* = 0.20, Fig. 1C). In addition, there are no significant differences in the serum level of testosterone (T), follicle-stimulating hormone (FSH) or luteinizing hormone (LH) between *Pnpla5*^*−/−*^ and *Pnpla5*^+*/*+^ male rats either (Fig. 1D). The calculated *P*-value of these hormones was 0.16, 0.43 and 0.11, respectively. Interestingly, the TG content of testes was no significantly different (*P* = 0.36, Fig. S 2), despite *Pnpla5* knockout resulted in increased serum TG levels [3].

**Fig. 2:**
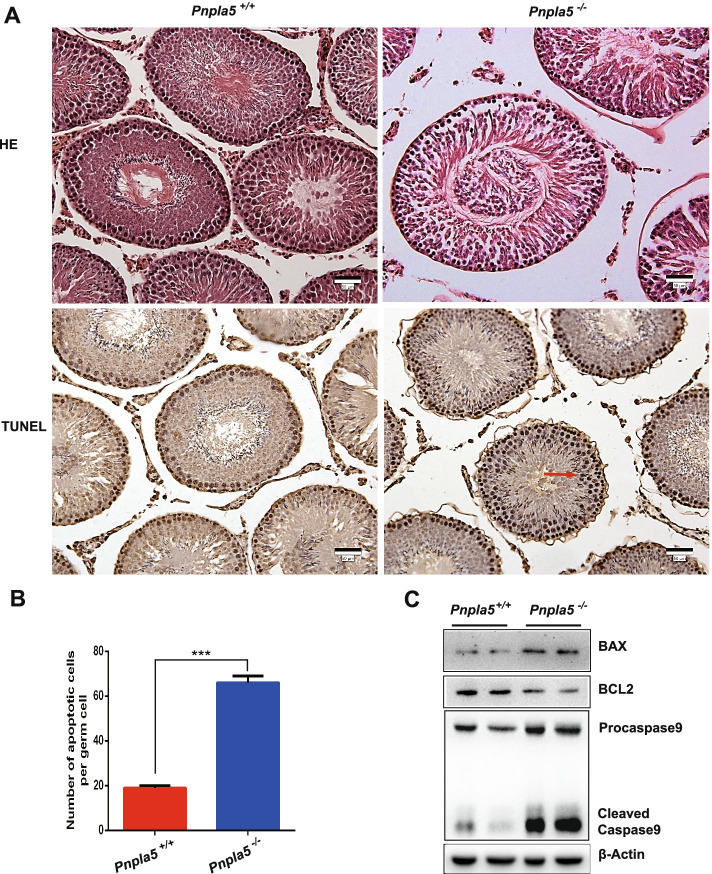
HE and TUNEL staining of testes and apoptotic protein expression in *Pnpla5*^*−/−*^ and *Pnpla5*^+*/*+^ male rats. **A** HE and TUNEL staining of testicular tissue. *Pnpla5*^*−/−*^ testes appeared loose, disarranged histological structure, with detached epithelial cells. Red arrows indicate apoptotic cells. Scale bar = 50 μm. The TUNEL staining slides were counterstained with hematoxylin. **B** The number of apoptotic cells was significantly increased in testicular tissue of *Pnpla5*^*−/−*^ male rats. *** *P* < 0.001. **C** The expression levels of apoptosis-related proteins in the testicular tissue were measured by western blotting. β-actin was used as the internal reference. All data are presented as means ± standard deviation (SD)

### Knockout of *Pnpla5* induces cell apoptosis in testicular tissue of rats

Previously, our group reported that *Pnpla5* KO significantly reduce the fertility of male rats [9]. Compared to the *Pnpla5*^+*/*+^ *rats,* the impregnating rate, and sperm cell curvilinear velocity and progressive motility percentage of their KO counterparts, were significantly decreased. Additionally, *Pnpla5*^*−/−*^ testes appeared loose, and had an disarranged histological structure, with detached epithelial cells (Fig. 2A) [9]. To further determine the *Pnpla5* gene’s role in testicular apoptosis regulation, TUNEL staining and western blotting assays were performed. The results showed that *Pnpla5* deletion caused an abnormal increase in the number of apoptotic cells in the rat testicular tissue (Fig. 2A). Statistically, the number of apoptotic cells were expected to significantly increase (Fig. 2B, *P* < 0.001). In addition, the expression levels of pro-apoptotic genes like *Bax*, *Caspase 9* increased while those anti-apoptotic genes, such as *Bcl2,* decreased (Fig. 2C). This is consistent with the TUNEL assay results. The mRNA expression level of *Nlrp3* was also measured using quantitative PCR (qPCR), and no significant differences were found (Fig. S3).

### *Pnpla5* KO significantly influences the expression level of proteins involved in steroid metabolism and wound healing process

To explore the effect of *Pnpla5*^*−/−*^ on spermatogenesis and sperm maturation at the proteomic level, we used gel-free iTRAQ and liquid chromatography-tandem mass spectrometry (LC–MS/MS) quantitative proteomic analyses to compare *Pnpla5*^*−/−*^ with *Pnpla5*^+*/*+^ rat testes. With false discovery rate (FDR) < 1.0% and specific peptides identified per protein ≥ 2, a total of 5118 proteins were identified using the UniProt rat protein database. Proteins that had an absolute value of log2 of KO/WT ≥ 0.26 and *P-*values < 0.05 (via *t-*test) were identified as differentially expressed proteins (DEPs). Forty nonredundant DEPs were identified in *Pnpla5*^*−/−*^ rat testis (Table 1), of which 35 were down-regulated and 5 were up-regulated.

**Table 1:** Differentially expressed proteins in *Pnpla5*^*−/−*^ rat testis

Symbol	Entrez Gene ID	Accession Number	Description	Log2 (KO/WT)	Up-/Down-regulated
**Akr1c19**	**307096**	**D3ZEL2_RAT**	**Aldo–keto reductase family 1, member C19**	**-1.34**	**down**
LOC100361706	100361706	LAC2_RAT	Lambda-chain C1-region-like	-0.67	down
Olfml1	361621	OLFL1_RAT	Olfactomedin-like protein 1	-0.57	down
Fmod	64507	FMOD_RAT	Fibromodulin	-0.56	down
Lgals5	25475	G3V7N2_RAT	Galectin	-0.55	down
Eng	497010	M0RA19_RAT	Endoglin	-0.55	down
Ogn	291015	D3ZVB7_RAT	Osteoglycin	-0.45	down
C6	24237	CO6_RAT	Complement component C6	-0.44	down
**Hsd3b3**	**682974**	**3BHS2_RAT**	**Hydroxy-delta-5-steroid dehydrogenase, 3 beta- and steroid delta-isomerase 3**	**-0.43**	**down**
F10	29243	A0A0H2UHR6_RAT	Coagulation factor X	-0.41	down
Smarcd1	363002	D3ZBS9_RAT	SWI/SNF related, matrix associated, actin dependent regulator of chromatin, subfamily d, member 1	-0.41	down
Kng1	288001	F7EUK4_RAT	Kininogen 1	-0.4	down
Calb2	117059	CALB2_RAT	Calretinin (CR)	-0.37	down
Igg-2a	367586	IGG2A_RAT	Ig gamma-2A chain C region	-0.37	down
Rcn3	494125	A0A0G2K022_RAT	Reticulocalbin 3	-0.35	down
**Apoa2**	**25649**	**APOA2_RAT**	**Apolipoprotein A2**	**-0.34**	**down**
Ttc14	310314	D3ZM05_RAT	Tetratricopeptide repeat domain 14	-0.32	down
Lum	81682	LUM_RAT	Lumican	-0.3	down
Mettl26	302998	CP013_RAT	Methyltransferase-like 26	-0.3	down
Spata5l1	691729	D4A2B7_RAT	Spermatogenesis-associated 5-like 1	-0.3	down
**Pon1**	**84024**	**PON1_RAT**	**Paraoxonase 1**	**-0.29**	**down**
**Hsd17b3**	**117182**	**DHB3_RAT**	**Hydroxysteroid (17-beta) dehydrogenase 3**	**-0.29**	**down**
Cpn2	303861	F1LQT4_RAT	Carboxypeptidase N subunit 2	-0.29	down
RT1-CE11	414791	HA12_RAT	RT1 class I histocompatibility antigen, AA alpha chain	-0.29	down
Paox	293589	D3Z8W0_RAT	Polyamine oxidase	-0.28	down
LOC299282	299282	A0A0G2K9B1_RAT	Serine protease inhibitor A3N	-0.28	down
Gpatch11	362685	F1LV52_RAT	G patch domain-containing 11	-0.28	down
Arid3b	367092	D3ZGC2_RAT	AT rich interactive domain 3B (Bright like) (Predicted) (AT-rich interaction domain 3B)	-0.28	down
Dcn	29139	PGS2_RAT	Decorin	-0.27	down
Nfib	29227	O70185_RAT	Nuclear factor I/B	-0.27	down
Galk2	296117	GALK2_RAT	N-acetylgalactosamine kinase	-0.27	down
Hspa12b	311427	D3ZVM5_RAT	Heat shock protein family A (Hsp70) member 12B	-0.27	down
Mlph	316620	G3V8M0_RAT	Melanophilin	-0.27	down
Lemd2	361807	Q4KM57_RAT	LEM domain-containing 2	-0.27	down
LOC100134871	100134871	HBB2_RAT	Hemoglobin subunit beta-2	-0.27	down
Ndufa5	25488	NDUA5_RAT	NADH:ubiquinone oxidoreductase subunit A5	0.27	up
Mydgf	501282	M0R3V4_RAT	Myeloid-derived growth factor	0.27	up
Hmgn5	681284	HMGN5_RAT	High mobility group nucleosome-binding domain-containing protein 5	0.27	up
Aldh1a7	29651	AL1A7_RAT	Aldehyde dehydrogenase family 1 member A7)	0.28	up
Prpf39	314171	D4A5S9_RAT	Pre-mRNA-processing factor 39	0.29	up

*DEP*, differentially expressed protein, *KO/WT*, knockout/wild-type

Bold, DEPs that are involved in steroid metabolism

**Fig. 3:**
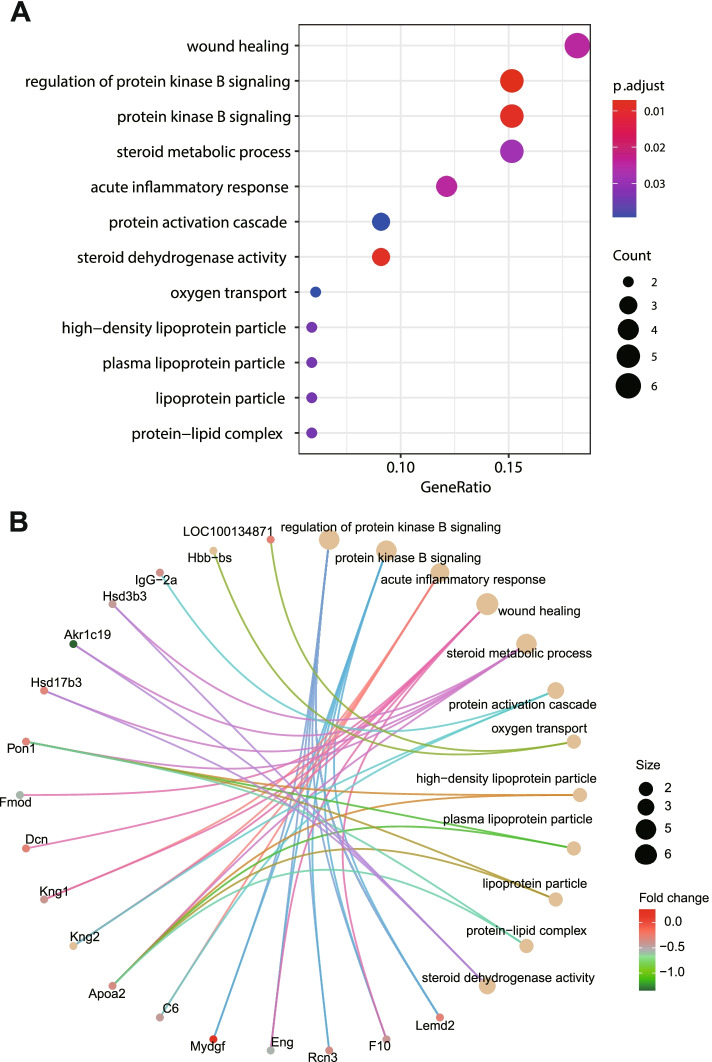
GO enrichment analysis of DEPs. **A** Dotplot of GO enrichment analysis for DEPs. GeneRatio: ratio of the number of differentially expressed genes to the number of total genes involved in the corresponding GO term; p.adjust: adjusted *P* value; Count: the number of genes that were enriched into the corresponding GO term. **B** Gene-concept network of GO enrichment analysis for DEPs. *Pon*1 and *Apoa*2 were involved in multiple GO terms. Size: gene number; Fold change: Log2 (KO/WT)

To explore the biological functions of these identified DEPs, Gene ontology (GO) and Kyoto Encyclopedia of Genes and Genomes (KEGG) enrichment analysis were performed. The GO enrichment analysis (Fig. 3A, Table S1) showed the DEPs significantly enriched in the following GO terms; wound healing, protein kinase B signaling, steroid metabolic process, acute inflammatory response, protein activation cascade and oxygen transport. Interestingly, several steroid metabolism-related GO terms were enriched, including steroid metabolic process (biology process class), steroid dehydrogenase activity (molecular function class), and protein-lipid complex (cellular component class). This enriched ‘protein-lipid complex’ is also the parental term of high-density lipoprotein particles, plasma lipoprotein particles and lipoprotein particles. These terms were enriched by GO enrichment analysis and are highly correlated with the abnormal lipid level found in *Pnpla5*^*−/−*^ rats [3], indicating that *Pnpla5* interacts with genes enriched in these terms to regulate steroid metabolism in rats. DEPs, such as *Apoa2* (Apolipoprotein A2)*, Pon1* (Paraoxonase 1)*, Hsd17b3* (Hydroxysteroid (17-beta) dehydrogenase 3)*, Akr1c19* (Aldo–keto reductase family 1, member C19) and *Hsd3b3* (Hhydroxy-delta-5-steroid dehydrogenase, 3 beta- and steroid delta-isomerase 3) are involved in these steroid metabolic processes, and are all down-regulated in *Pnpla5*^*−/−*^ rat testis (Table 1). Using gene-concept network analysis, we depicted the linkages of DEPs and enriched GO terms (Fig. 3B). *Apoa2* and *Pon1* were enriched into multiple GO terms, and may play key roles in the process of lipids metabolism and wound healing. Further studies on these genes could uncover the underlying mechanisms of the phenotypic abnormalities induced by *Pnpla5* KO.

**Fig. 4:**
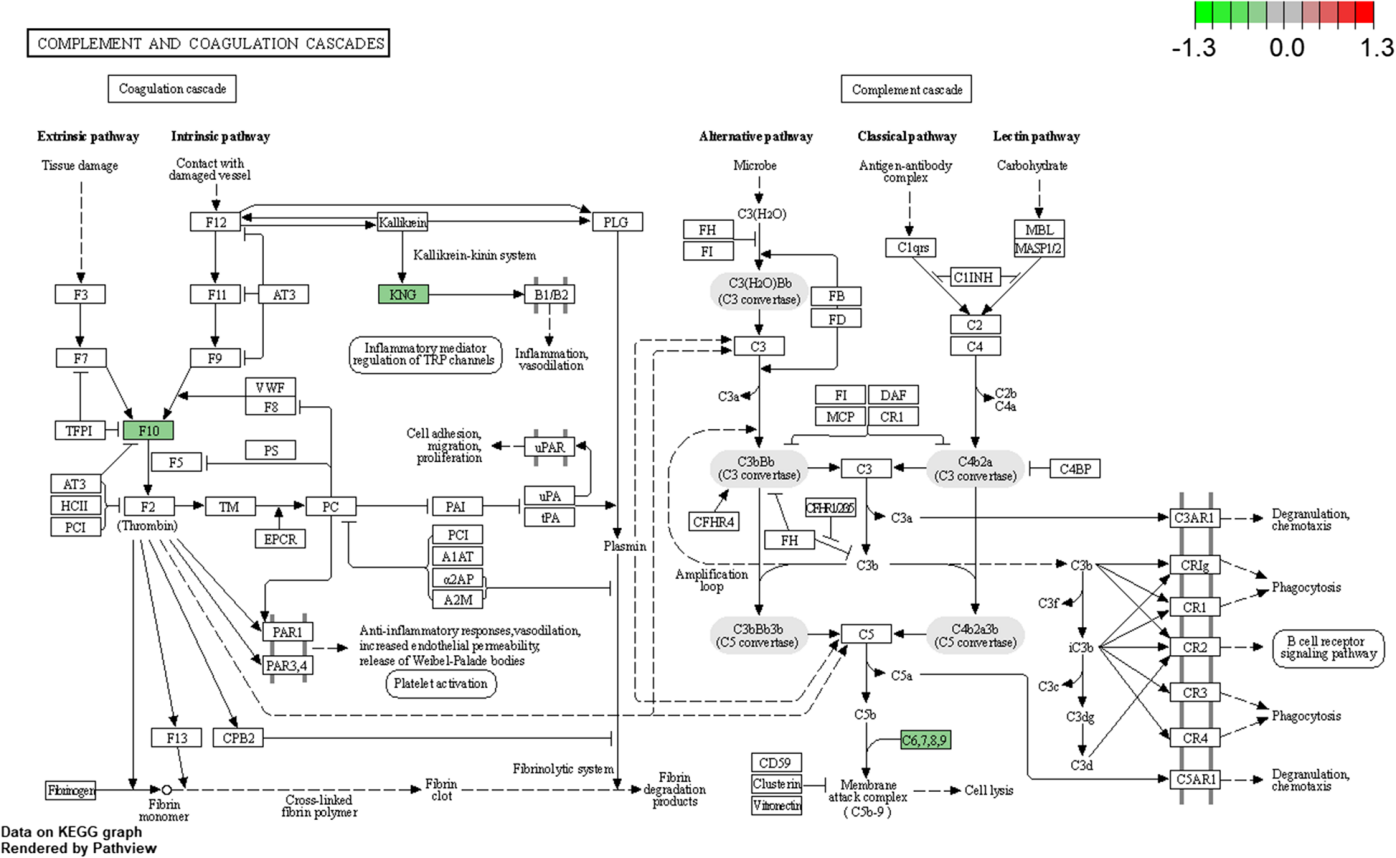
F10, C6 and KNG were enriched in complement and coagulation cascades pathways (modified from an image obtained by KEGG [15–17]). Proteins down-regulated in *Pnpla5*^*−/−*^ rats are highlighted in green. Red arrows indicate activation, and the dotted red arrow indicates indirect activation

Previously, we reported uncontrollable bleeding and a longer blood coagulation time in *Pnpla5*^−/−^ rats [3]. GO enrichment analysis revealed that several DEPs were enriched for wound healing and acute inflammatory response terms (Fig. 3A, Table S1), including *Dcn* (Decorin), *Fmod* (Fibromodulin), *F10* (Coagulation factor X), *Eng* (Endoglin), *C6* (Complement C6), *Apoa2*, *Kng1* (Kininogen 1), and *Kng2* (Kininogen 2). *Dcn, Fmod, Kng2, Kng1, F10,* and *Eng* were enriched in the wound healing GO term and *C6, Apoa2, Kng1,* and *Kng2* were enriched in the acute inflammatory response GO term. Moreover, *C6/Kng1/Kng2 and F10* were also enriched in the complement and coagulation cascades pathways (Fig. 4). F10, C6, and KNG play essential roles in wound healing and acute inflammatory response. F10 can activate F2 (also known as thrombin) [12], which indirectly interacts with the C5 precursor, which is proteolytically processed to generate C5b. One unit of complements 5b, 6, 7, and 8 and several units of complement 9 form a membrane attack complex (MAC) [13] (Fig. 4). MAC can cause cell lysis and death. It forms trans-plasma membrane channels on the surface of pathogenic bacteria [13], neutralizing their threating. KNG is essential for blood coagulation and assembly of the kallikrein-kinin system which is involved in hemostasis [14]. Moreover, F10, C6 and KNG were down-regulated in *Pnpla5*^−/−^ rats, which blocked MAC-induced cell lysis and inhibited hemostasis. Therefore, *Pnpla5* KO induced down-regulation of F10, C6, and KNG may contribute to the abnormal bleeding in *Pnpla5*^−/−^ rats. Further biological and clinical studies are required to fully elucidate the underlying molecular mechanism.

## Discussion

Although *Pnpla5* is minimally expressed in rat tissues [3], a variety of phenotypic abnormalities arise from its absence. Lipid metabolism disorders, abnormal bleeding, and reduced male fertility induced by *Pnpla5* deletion have been reported [3, 9, 18]. In this study, we further explored the effect of *Pnpla5* knockout on male rats to augment the knowledge of this gene’s biological functions.

The body and reproductive organs weight and serum hormone levels of *Pnpla5*^*−/−*^ and *Pnpla5*^+*/*+^ rats were measured. No significant differences were identified between the two groups (Fig. 1), despite Vendramini et al. (2014) reporting that testicular weight loss in male rats can be caused by lipid metabolism disorder [10]. Meanwhile, the relative weight (ratio of tissue to body weight) of testicular tissue was significantly higher in *Pnpla5*^*−/−*^ rats, necessitating the further study of underlying mechanism. Moreover, quantitative proteomic analysis revealed that genes involved in the steroid metabolic processes and protein-lipid complexes (including high-density lipoprotein particles, plasma lipoprotein particles and lipoprotein particles) were significantly enriched. This is consistent with a previous report that *Pnpla5*^*−/−*^ rats have abnormal serum TC, TG, HDL-C and LDL-C levels, indicating that *Pnpla5* may play an important role in protein-lipid complex formation.

Quantitative proteomic analysis revealed several genes and potential pathways that may be involved in the molecular mechanisms underlying abnormal bleeding in *Pnpla5*^*-/-*^ rats. *Dcn, Fmod, Kng2, Kng1, F10, C6, Apoa2,* and *Eng* were enriched in the wound healing and acute inflammatory response GO terms, and may contribute to the uncontrollable bleeding and longer blood coagulation time observed in *Pnpla5*^*-/-*^ rats [3]. Notably, *F10, C6, Kng1,* and *Kng2* were also enriched in the complement and coagulation cascades pathway by KEGG enrichment. The down-regulation of *F10/C6* directly blocks MAC formation [12], which is crucial for neutralizing bacteria [13]. Therefore, it is reasonable to assume that *Pnpla5*^*-/-*^ rats may also suffer from recurrent bacterial infections.

Previous reports noted the decreased pregnancy rates in female rats that mated with *Pnpla5*^*−/−*^ male rats, indicative of reduced fertility and sperm motility, and supported by the disarranged histological structure of *Pnpla5*^*−/−*^ male rats testes [9]. In this study, apoptosis in the testicular tissue was identified by TUNEL assay (Fig. 2), and confirmed by western blotting of apoptosis related proteins. A high-fat diet not only alters the testicular morphology, with reduced seminiferous epithelium height and tubule diameter but it also decreases the proliferation of spermatogenic cell [11]. Therefore, the disorders of lipid metabolism caused by *Pnpla5* knockout may be one of the reasons for abnormalities in testicular tissue.

In summary, *Pnpla5*^*−/−*^ rats were used as the animal model to study the biological functions of *Pnpla5*. The data showed that this gene’s deletion in rats caused no significant difference in body, testicular or epididymal tissue weight, or hormones levels. However, *Pnpla5* KO significantly increases testicular apoptosis level, indicating that the deletion of *Pnpla5* adversely affects the reproductive organs. Moreover, iTRAQ proteomic analysis revealed that DEPs, involved in steroid metabolism and wound healing, were significantly decreased in *Pnpla5*^*−/−*^ rats. Our study sheds new light, but does not fully address, the underlying mechanisms of phenotypic abnormalities induced by *Pnpla5* knockout. Several questions remain unanswered at present. The fact that, *Pnpla5* deletion had no effect on the TG content of testes, yet increased the TG serum levels, is a discrepancy that needs to be resolved in the future. Much work is yet to be done to fully characterize the biological functions of the *Pnpla5* gene.

## Materials and methods

### Animals

The *Pnpla5* KO SD rats used in this study were obtained as previously described [3]. All rats were 3-month-old and specific pathogen-free (SPF) and were housed in standard cages and provided with food and water, ad libitum. All animal experiments performed in this study were approved by the Animal Care and Use Committee of the Institute of Animal Sciences, Chinese Academy of Agricultural Sciences (IAS2017-8).

### Serum hormone analysis

After pentobarbital sodium (Sigma-Aldrich, Missouri, USA) anesthesia (15–40 mg/kg, intraperitoneal injection), blood samples were collected from the orbital of *Pnpla5*^*−/−*^ and *Pnpla5*^+*/*+^ male rats. Whole blood was centrifuged at 4000 × g for 10 min at room temperature to obtain the serum. Then serum testosterone, follicle-stimulating hormone, and luteinizing hormone levels were measured using a Hitachi 7080 Biochemical Automatic Analyzer (Hitachi, Tokyo, Japan).

### qPCR

Total RNA was extracted from testes of rats using TRIzol (Invitrogen, California, USA), and reverse transcribed to cDNA using the RevertAid First Strand cDNA Synthesis kit (Thermo Fisher Scientific, Massachusetts, USA). qPCR was performed using TB Green® Premix Ex Taq II (TaKaRa, Tokyo, Japan) following the manufacturer’s instruction. *Gapdh* was used as a control, and the relative gene expression level was calculated using the 2 ^− ΔΔCt^ method. The primers used are listed in Table S3.

### Tissue TG Content Assay

The TG content in the *Pnpla5*^*−/−*^ and *Pnpla5*^+*/*+^ testes were measured by a Tissue TG Content Assay Kit (Cat. No. E1013), following the manufacturer’s instructions.

### Western blotting

For western blotting, the tissues were homogenized, and the supernatants were collected after centrifugation. Total protein concentration was quantified using a BCA kit (Beyotime, Shanghai, China). Equal amounts of total protein were separated by 10% sodium dodecyl sulfate–polyacrylamide gel electrophoresis (SDS_PAGE, EpiZyme, Shanghai, China) and transferred to a polyvinylidene difluoride membrane (Millipore, Massachusetts, USA). After blocking with 5% non-fat milk at 4 °C overnight, the membrane was incubated with primary antibodies at 4 °C overnight, washed three times with Tris-buffered saline with tween 20 (TBST) buffer (EpiZyme, Shanghai, China), and then incubated with secondary antibodies at room temperature for 2 h. The membrane was washed three times with TBST buffer, and the blots were stained using an enhanced chemiluminescence (ECL) method on a Tanon-5200 Chemiluminescent Imaging System and ImageJ (National Institutes of Health, Maryland, USA). Immunoblotting used the following primary antibodies: BAX (Cat. No. 2772, Cell Signaling Technology, Massachusetts, USA), BCL2 (Cat. No. Ab182858, Abcam, Cambridge, UK), anti-caspase 9 (Cat. No. 9508, Cell Signaling Technology, Massachusetts, USA), and β-actin antibody (Cat. No. 4970 s, Cell Signaling Technology, Massachusetts, USA). We used anti-rabbit IgG (Cat. No. 7074, Cell Signaling Technology, Massachusetts, USA) as the secondary antibody.

### Histological analysis of the testis

Fixed rat testis was embedded in paraffin in vertical position. Then embedded testis-tissue was sliced with a microtome into 5‐7 µm thick sections and adhered to the slides. After incubated in a xylene bath and rehydration, the slides were stained with hematoxylin and eosin (HE). These HE stained slides were used for morphological analysis of the testicular structure.

### TUNEL assay

To evaluate the status of the testis cells, we preformed TUNEL assays, using the In Situ Cell Death Detection Kit (Cat. No. 11684817910, Roche, Basel, Switzerland). Briefly, testicular tissue samples from *Pnpla5*^*−/−*^ and *Pnpla5*^+*/*+^ rats were fixed in 4% formaldehyde for 24 h and embedded in paraffin. Then, 5‐7 μm paraffin sections were adhered to the slides. These section slides were incubated in a xylene bath, at room temperature, for 10 min. The slides were then rehydrated by being transferred through a graded ethanol series: 3 min in 90, 80, and 70% ethanol, each, followed by 3 min in double-distilled water. The sections were covered by pipetted proteinase K solution (20 mg/mL) and incubated for 15 min at room temperature. Thereafter, the slides underwent two 5-min PBS washing cycles. The sections were then covered with 50 µL TUNEL reaction buffer and converter-POD at 37 °C, for 1 h and 30 min, respectively, as well as 50 µL IDAB substrate for 10 min at room temperature. Thereafter, the slides were washed with PBS and counterstained with hematoxylin for 10 min. Then stained slides were then subjected to a decreasing alcohol series. After incubation for 20 min in xylene, the slides were mounted. Tissue sections were examined by microscopy. At least ten fields were randomly selected for each sample, the number of TUNEL-positive cells in each field was counted, and the average number of apoptotic cells in each sample was calculated.

### Protein extraction, digestion, and labeling with iTRAQ reagents

For the proteomics quantitative analysis, two groups of 3-month-old rats (n = 3), *Pnpla5*^+*/*+^ and*Pnpla5*^*−/−*^ were analyzed. The rats were euthanized by cervical dislocation under deep anesthesia, and the testes were recovered and stored in liquid nitrogen. Total protein was isolated as previously described [19]. Protein concentrations were measured by the Bradford assay using bovine serum albumin (BSA) as the standard. For trypsin digestion and iTRAQ labeling, 200 µg of protein from each sample was reduced with 25 mM DTT (Bio-Rad, California, USA) at 37 °C for 1 h, alkylated with 50 mM iodoacetamide (Bio-Rad, California, USA) for 30 min at room temperature in the dark, and then digested with 4 µg trypsin (Promega, Wisconsin, USA) at 37 °C overnight. The digested samples were labeled with iTRAQ reagents according to the manufacturer’s instructions. In brief, labels 114–116 and labels 117–119 were used for the relative abundance of three *Pnpla5*^*−/−*^ and *Pnpla5*^+*/*+^testicular tissues, respectively. The labeled samples were then mixed and vacuum-dried.

### LC–MS/MS analysis, peptide identification and quantification

The dried peptides were reconstituted in 10 µL of 0.1% formic acid and loaded onto a low-pH reversed-phase C18 column (75 μm × 100 mm, 3 μm), interfaced with an Orbitrap Q-Exactive-plus (Thermo Fisher Scientific, Massachusetts, USA). Three independent biological replicates were performed, and the resulting peptides were independently analyzed using LC–MS/MS. The raw data files, produced by mass spectrometry (MS) and protein quantification, were identified using Mascot (Version 2.5.1) search against the rat protein database (downloaded from UniProt). The parameters used for database searching were as follows: trypsin selected as the enzyme, a maximum of two missed cleavages were allowed, precursor mass tolerance was set to 10 ppm, fragment mass tolerance was set to 0.05 KDa, carbamidomethylation of cysteine was set as a fixed modification, and methionine oxidation and acetyl at the N-terminal and lysine side chains were allowed as dynamic modifications. The FDR was set to 1% for protein identification. Proteins with *P* < 0.05 and absolute value of log2 of KO/WT ≥ 0.26 were considered DEPs.

### Bioinformatics analysis

To better understand the biological functions of DEPs, Gene Ontology (GO) and Kyoto Encyclopedia of Genes and Genomes (KEGG) enrichment analyses were performed using the clusterProfiler (version 4.2.1) R package [20]. GO terms with a corrected *P* < 0.05 were considered significantly enriched by differential expressed genes. KEGG pathways with corrected *P* < 0.05 were considered significantly enriched. GO and KEGG enrichment results were visualized using the ggplot2 (version 3.3.5), pathview (version 3.1.4) [21], and ggpubr (version 0.4.0) R packages.

### Statistical analysis

All data are presented as means ± standard deviation (SD). Data from the two groups were compared using an unpaired t-test. Data were analyzed using GraphPad Prism version 8 (GraphPad Software, California, USA). *P* values less than 0.05, 0.01, and 0.001 are indicated by *, **, and ***, respectively. Each rat served as an experimental unit.

## Supplementary Information

**Additionalfile 1: Figure S1. **The original western blotting gels of Figure 2C. **Figure S2.** Triglyceride content of *Pnpla5*^*+/+*^ and *Pnpla5*^*-/-*^rat testes. **Figure S3.** The *Nlrp3* expression level in testes.

**Additionalfile 2: Table S1.** GO enrichment result.

**Additionalfile 3: Table S2.** KEGG enrichment result.

**Additionalfile 4: Table S3.** qPCR primers.

## Data Availability

Upon reasonable request, the corresponding author will share the data supporting this study.

## Abbreviations

KO: Knockout
WT: Wild-type
TG: Triglyceride
LDL-C: Low density lipoprotein cholesterol
TC: Total cholesterol
HDL-C: High density lipoprotein cholesterol
TUNEL: Terminal deoxynucleotidyl transferase-mediated dUTP nick end labeling
iTRAQ: Isobaric tags for relative and absolute quantitation
T: Testosterone
FSH: Follicle-stimulating hormone
LH: Luteinizing hormone
FDR: False discovery rate
DEP: Differentially expressed protein
GO: Gene ontology
KEGG: Kyoto Encyclopedia of Genes and Genomes
MAC: Membrane attack complex
