# Supplementary material for: Pnpla5-knockout rats exhibit reduced expression levels of proteins involved in steroid metabolism and wound healing compared to wild-type rats
Source: BMC Genomics. 2022 Aug 12;23:583. doi: 10.1186/s12864-022-08835-8 (PMC9375266; doi:10.1186/s12864-022-08835-8)

**Figure S1** The original western blotting gels of Figure 2C.


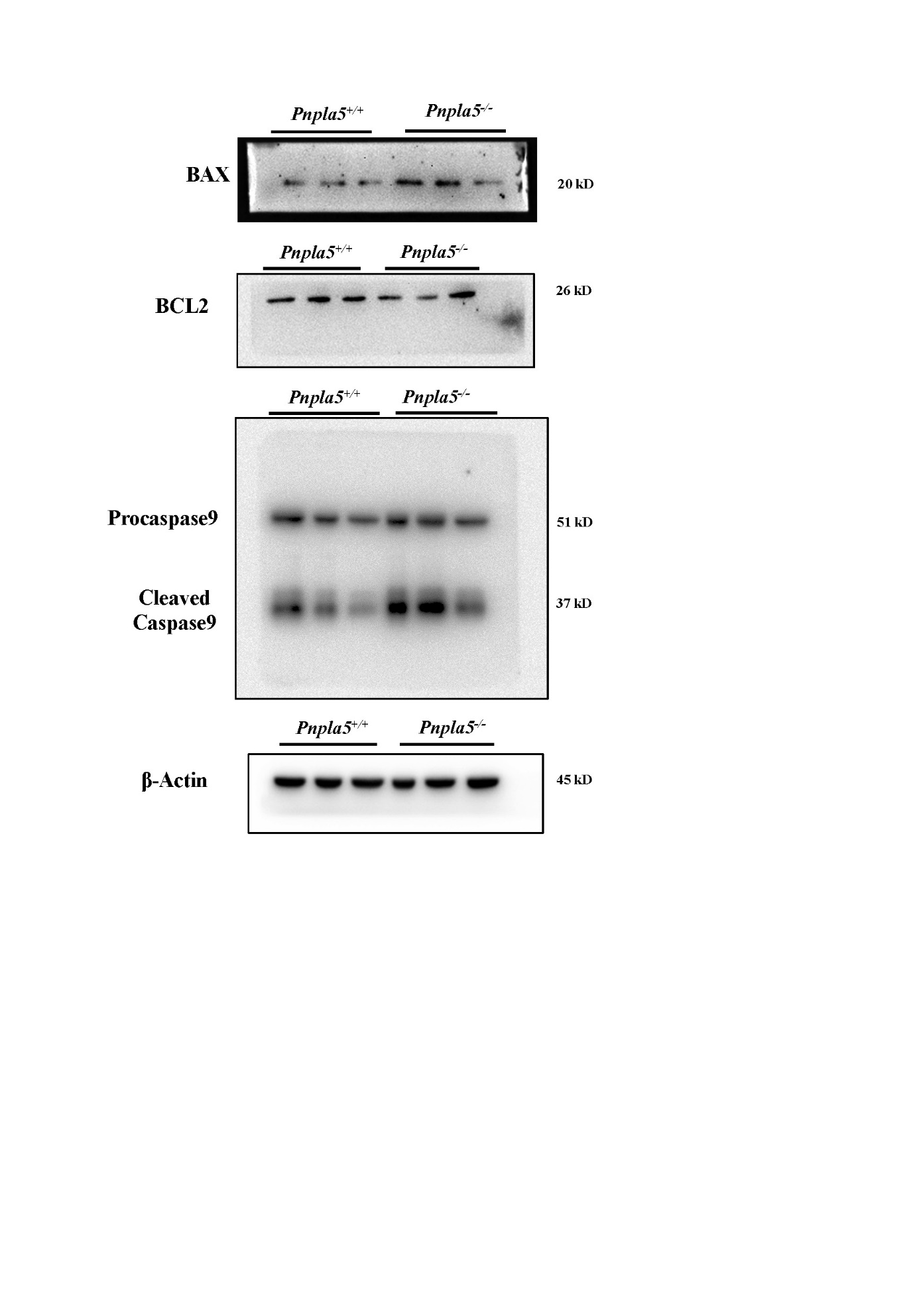


**Figure S2** Triglyceride content of *Pnpla5^+/+^* and *Pnpla5^-/-^* rat testes.


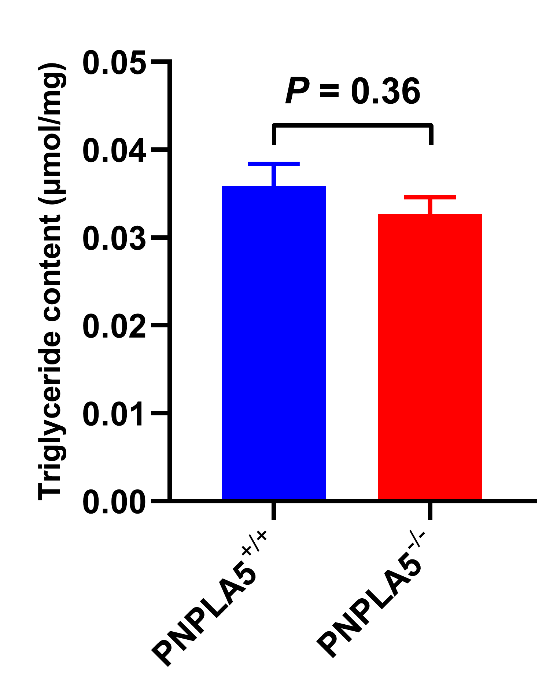


**Figure S3** The *Nlrp3* expression level in testes.


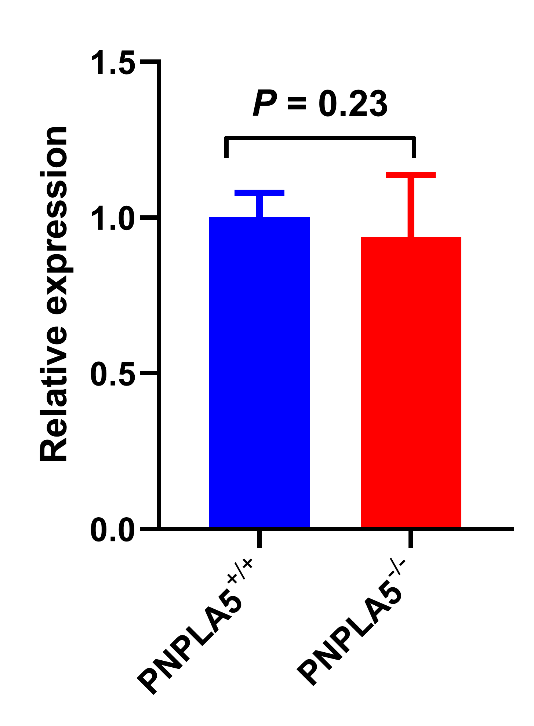

Supplement: Supplementary file 1 — Additionalfile 1: Figure S1. The original western blotting gels of Figure 2C. Figure S2. Triglyceride content of Pnpla5+/+ and Pnpla5-/-rat testes. Figure S3. The Nlrp3 expression level in testes. [file 12864_2022_8835_MOESM1_ESM.docx]
